# Long-term trends in Alzheimer’s disease and other dementias deaths with high body mass index in China from 1990 to 2019, and projections up to 2042

**DOI:** 10.1186/s13690-024-01273-w

**Published:** 2024-03-26

**Authors:** Mengjun Tao, Hao-Yang Guo, Xincan Ji, Wei Wang, Hui Yuan, Hui Peng

**Affiliations:** 1 Health management center, The First Affiliated Hospital of Wannan Medical College, Wuhu, Anhui China; 2 School of Public Health, Wannan Medical College, Wuhu, Anhui China; 3 Department of Science and Technology, The First Affiliated Hospital of Wannan Medical College, Wuhu, Anhui China

**Keywords:** Alzheimer’s disease, Dementia, Mortality, Joinpoint regression, Age-period-cohort, Trend-analysis, Prediction

## Abstract

**Background:**

In China, the rising prevalence of high Body Mass Index (BMI) is linked to increasing health issues, including Alzheimer’s disease (AD). This study analyzes mortality trends related to AD and other dementias associated with high BMI from 1990 to 2019, considering age, period, and birth cohort effects, and forecasts future trends.

**Methods:**

We analyzed mortality data for AD and other dementias linked to high BMI in Chinese residents from the Global Burden of Disease 2019 database. Using Joinpoint regression, we examined age-standardized mortality rate (ASMR) trends and calculated annual and average annual percentage changes (APC and AAPC). Age-period-cohort models provided deeper insights, with Bayesian models used to project future ASMR trends to 2042.

**Results:**

From 1990 to 2019, the ASMR for AD and other dementias associated with high BMI in China showed an overall increasing trend. Females had a lower increase rate than males, yet their overall levels remained higher. Specifically, the ASMR for males increased by an average of 2.70% per year, peaking between 2006 and 2010, while for females, it increased by an average of 2.29% per year, also peaking in the same period. Age-period-cohort analysis revealed increasing mortality relative risk with age and period, but a decrease with birth cohort. Projections suggest a continued rise in ASMR by 2042, with rates for males and females expected to be 2.48/100,000 and 2.94/100,000, respectively.

**Conclusion:**

The increasing mortality trend from AD and other dementias associated with high BMI highlights the urgent need for policy interventions focused on overweight prevention, particularly vital for addressing the health challenges in China’s aging population.

**Supplementary Information:**

The online version contains supplementary material available at 10.1186/s13690-024-01273-w.

**Table:** 

Text box 1. Contributions to the literature
• The Age-period-cohort model can independently analyze the effects of age, period, and cohort on disease risk.
• The study predicts that ASMR will increase more rapidly for male residents of China than for females from 2020 to 2042, providing policymakers with valuable information about future health trends.
• As China’s population ages and the prevalence of high BMI rises, implementing early interventions and prioritizing preventive measures for Alzheimer’s Disease (AD) has become increasingly critical.

## Background

China, the most populous country in the world, is facing the challenge of an aging population. In 2021, it was reported that there were 267 million elderly individuals aged 60 or older in China, accounting for 18.9% of the total population. The ongoing trend of an aging population in China has led to an increase in the burden of neurological diseases, particularly dementia. Alzheimer’s disease (AD) is the most common type of dementia, with AD constituting 60–80% of all dementia cases globally [1]. A cross-sectional study conducted in China revealed that approximately 9.83 million individuals aged 60 or older are affected by AD, corresponding to a prevalence rate of 3.9% (95% *CI* [3.8 − 4.1%]) [2].

Various factors contribute to the development of AD, one of which is high body mass index (BMI), a modifiable influence factor [3]. High BMI, widely used as an indicator of obesity, has been associated with AD, although this relationship remains controversial [4]. Studies have indicated that a higher BMI in late life might be associated with a reduced risk of AD compared to those maintaining normal weight [5]. Conversely, individuals who experience a decrease in BMI from midlife to late life are associated with an increased risk of AD, with a hazard ratio of 1.20 (95% *CI* [1.09–1.33]) [6]. Furthermore, a meta-analysis showed that middle-aged individuals with a higher BMI, compared to a control group, have a hazard ratio of 1.33 (95% *CI* [1.03, 1.62]) for AD risk. In contrast, late-life high BMI is inversely correlated with AD risk, as indicated by a hazard ratio of 0.57 (95% *CI* [0.47, 0.68]) [7]. Although a direct causal relationship between high BMI and AD has not been conclusively established, epidemiological investigations suggest that high BMI may increase the risk of dementia under certain conditions [8]. Unhealthy diets, such as those high in sugar and fat, can lead to insulin resistance, which may impair brain functions and increase the risk of AD [9]. Furthermore, high BMI caused by such diets can result in chronic inflammation, which exacerbates neuroinflammation and cognitive decline associated with AD [10].

Gender differences significantly influence the connection between high BMI and AD. Variations in body fat distribution, metabolism, and hormones between women and men can alter AD’s risk factors and progression. For example, post-menopausal women experience increased insulin resistance related to high BMI due to decreased estrogen levels, which elevates their risk of AD [11]. Factors like brain volume, lipid metabolism, and inflammation might explain these gender-specific risks. Thus, it’s vital to include gender as a variable in BMI and AD studies.

As China confronts rapid population aging, the complex yet unclear relationship between AD and high BMI underscores the need for targeted research on its large and aging population. Limited epidemiological research on AD in China makes it challenging to obtain national-level information on the incidence and long-term trend of AD in individuals with high BMI. The objective of this study was to examine the long-term patterns in the mortality of AD and other dementias with high BMI in China from 1990 to 2019. This study also places a specific focus on potential gender differences. The findings from this study contribute to a better comprehension of the trends in high BMI and AD and other dementias in China, and investigated whether these trends could be attributed to periodic effects or cohort effects. Additionally, this study aimed to provide reference for the allocation of resources for the prevention of this disease.

## Materials and methods

### Data sources

This is an analytical epidemiological study using data from GBD 2019. Data on the association of high BMI with AD and other dementias in China, specifically the ‘Deaths’ data, were obtained from the Global Health Data Exchange (GHDx) section of the outcome tool (http://ghdx.healthdata.org/gbd-results-tool). Besides AD, this study also investigates other dementias, including but not limited to vascular dementia, HIV-associated dementia, and others. The data source provides comprehensive estimates of the risk-specific mortality for 87 mortality risk factors (including metabolic, environmental, occupational, and behavioral risks) between 1990 and 2019 and internally consistent estimates of age-specific all-cause and cause specific mortality for 369 diseases and injuries in 204 countries and territories, with the International Classification of Diseases 10th Revision (ICD-10) used to classify the diseases studied, making the estimates more accurate [12, 13]. Individuals aged 40 − 94 years of age were selected for this study. They were divided into 11 age groups using 5-year age categories. To avoid overlapping adjacent cohorts, six periods (1990–1994, 1995–1999, 2000–2004, 2005–2009, 2010–2014 and 2015–2019) were selected, and 16 birth cohorts were obtained by period minus age. The standardized population used in this study was derived from the world standard population compiled by GBD 2019, using a direct standardization method to age-standardize mortality from high BMI leading to AD and other dementias. High BMI in adults (age 18 + years) was defined as BMI ≥ 25 kg / m^2^ [14].

### Time trend analysis

Joinpoint regression models, recognized as a method for analyzing and illustrating trends in data over time and best fitting log non-linear models, were derived from the National Cancer Institute Surveillance Research Program (NCISRP). Using ‘time’ (year) as our independent variable and the metric of interest (e.g., incidence or prevalence rate) as the dependent variable, we utilized models to identify the optimal fit by evaluating the number and location of Joinpoints, employing the Monte Carlo permutation test for trend fitting [15]. The annual percentage change (APC) was calculated and the average annual percentage change (AAPC) in accordance with the methodologies outlined by the National Cancer Institute’s Joinpoint Regression Program [16]. The APC from year $y$ to $y+1$=$\frac{{R}_{y+1}-{R}_{y}}{{R}_{y}}$ ; AAPC $=\{\text{exp}\left(\frac{\sum {\omega }_{i}{b}_{i}}{\sum {\omega }_{i}}\right)-1\}\times 100$. For the ${i}^{th}$ segment with $i$ indexing the segments in the desired range of years, and ${\omega }_{i}$ as the length of each segment in the range of years. The confidence intervals (95% *CI*) for these metrics are provided in Supplementary Table 1. These methods align with the guidelines provided on the joinpoint analysis webpage [https://surveillance.cancer.gov/help/joinpoint/setting-parameters/method-and-parameters-tab/apc-aapc-tau-confidence-intervals/estimate-average-percent-change-apc-and-confidence-interval].

The APC was used to evaluate trends across time periods, and the AAPC was used to evaluate trends across time periods [17]. In Joinpoint software, APC confidence intervals are based on t-distributions. The AAPC confidence interval is based on the t-distribution when the number of connected points is 0 and on the normal distribution otherwise. The APC confidence intervals and the AAPC confidence interval were used to test whether there was a statistically significant trend change for each time period and the entire period.

### Age-period-cohort model

Because there is a linear relationship between age, period, and cohort, there can be problems with nonidentification, which makes it difficult to estimate unique sets of effects for each age, period, and cohort [18]. The age-period-cohort model is a widely used statistical method to analyze and understand the effects of age, period, and cohort on various health outcomes, including disease morbidity and mortality. This study used the intrinsic estimator (IE) algorithm to estimate the effect coefficients for age, period, and cohort, and the relative risk (RR) of mortality was obtained by natural log transformation [19]. The RR of mortality for a given age, period, or birth cohort, in comparison to each average level, is represented by the exponential of the effect coefficient. The fit of the model was assessed by the Akaike information criterion (AIC), Bayesian information criterion (BIC), and deviance [20].

### Prediction

This study used a Bayesian annual percentage change (BAPC) model to make predictions of standardized mortality in AD and other dementias with high BMI. In Bayesian inference, uncertainty about all unknown parameters is considered, and for this study, non-informative priors were employed [21]. The BAPC model is generally based on Poisson regression models, treating the age effect as a row variable, the period effect as a column variable, and the cohort effect as a cross-effect of the age effect and the period effect to construct a three-factor model of age, period, and cohort [22]. Integrated nested Laplacian approximation (INLA) was employed within the Bayesian age period cohort model to approximate the edge posterior distribution. This approach was specifically chosen to sidestep potential admixture and convergence issues that can arise with traditional Markov chain Monte Carlo (MCMC) sampling techniques for Bayesian methods.

### Statistical methods

Data collation was performed using Excel. Regression analysis was conducted using the Joinpoint regression program (version 4.9.0.0). Age-period-cohort model was constructed using Stata 17.0 software. The BAPC model, INLA, was carried out utilizing the BAPC and INLA packages in R (version 4.2.3) [23, 24].

## Results

### The age-standardized mortality rate (ASMR) for AD and other dementias among Chinese residents with high BMI

**Fig. 1:**
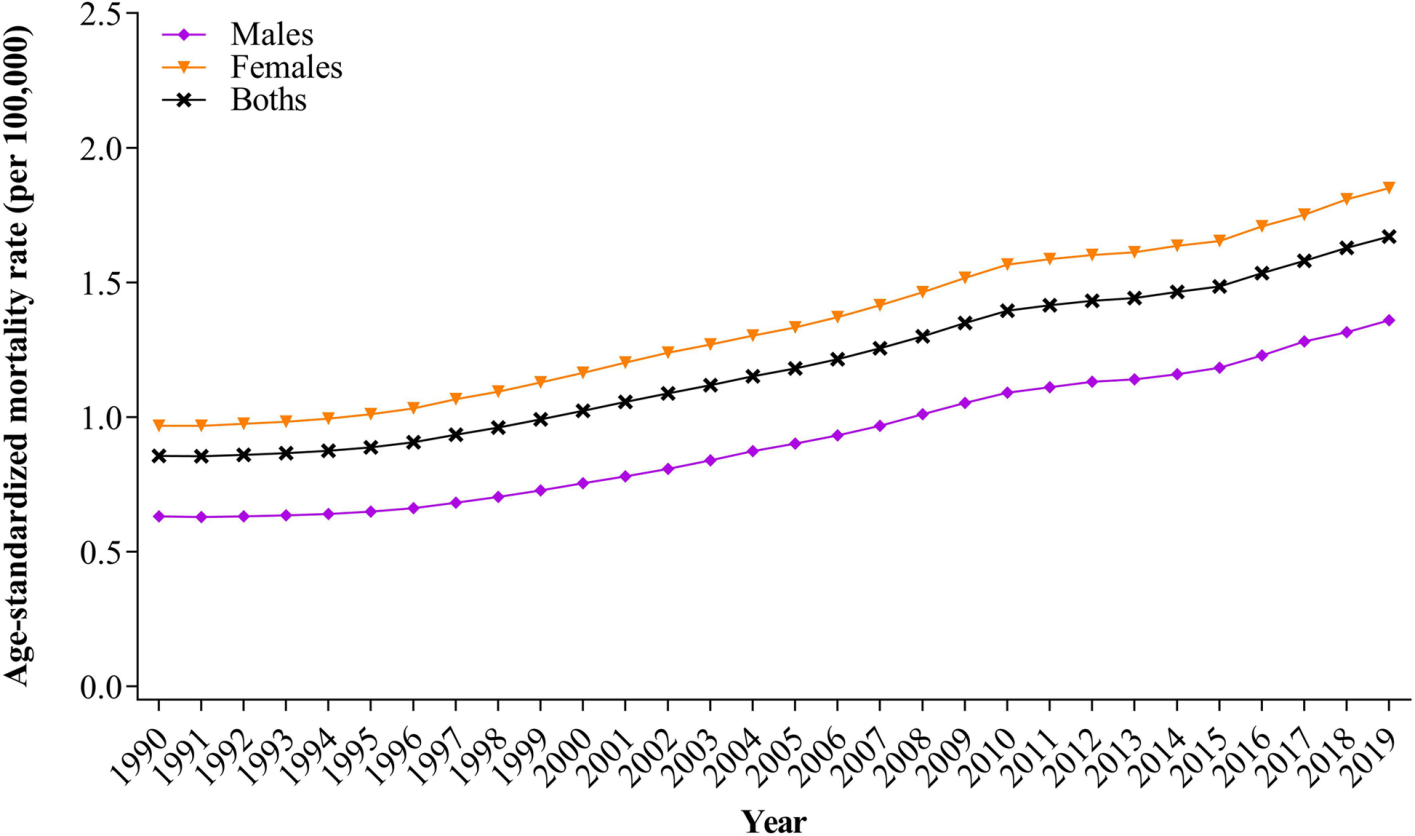
The trends of ASMR for AD and other dementias deaths with high BMI in China, 1990 − 2019

The ASMR for AD and other dementias with high BMI in China increased from 1990 to 2019. Notably, higher rates of increase were observed among females compared to males. Specifically, for males during this period, the ASMR increased by an average of 2.70% per year (AAPC = 2.70%, *CI* [2.56%, 2.84%], *P* = 0.008), with a peak between 2006 − 2010 (APC = 4.05%, *CI* [3.53%, 4.56%], *P* < 0.001). In contrast, for females from 1990 to 2019, it increased by an average of 2.29% per year (AAPC = 2.29%, *CI* [2.19%, 2.39%], *P* < 0.001), also peaking between 2006 − 2010 (APC = 3.42%, *CI* [3.02%, 3.83%], *P* < 0.001). The national best fitting log non-linear models for the entire population from 1990 to 2019 indicated an overall average ASMR increase of 2.35% per year (AAPC = 2.35%, *CI* [2.21%, 2.48%], *P* < 0.001), with a peak between 2007 − 2010 (APC = 3.60%, *CI* [2.76%, 4.44%], *P* < 0.001) (Fig. 1; Tables 1 and 2).

**Table 1:** The APC and AAPC of AD and other dementias deaths with high BMI in China, 1990 − 2019

Gender	Period	APC (%)	95%CI	* t*	* P*	AAPC (%)	95% CI	* Z*	* P*
“Male”-5 Joinpoints	1990–1993	0.16	-0.34 ~ 0.65	0.69	0.505	2.70	2.56 ~ 2.84	37.63	< 0.001
“Male”-5 Joinpoints	1993–1996	1.27	0.28 ~ 2.28	2.77	0.016				
“Male”-5 Joinpoints	1996–2006	3.55	3.45 ~ 3.64	83.62	< 0.001				
“Male”-5 Joinpoints	2006–2010	4.05	3.53 ~ 4.56	17.38	< 0.001				
“Male”-5 Joinpoints	2010–2015	1.61	1.29 ~ 1.92	11.03	< 0.001				
“Male”-5 Joinpoints	2015–2019	3.62	3.30 ~ 3.95	24.66	< 0.001				
“Female”-5 Joinpoints	1990–1995	0.84	0.66 ~ 1.01	10.25	< 0.001	2.29	2.19 ~ 2.39	47.06	< 0.001
“Female”-5 Joinpoints	1995–2002	3.04	2.90 ~ 3.18	48.78	< 0.001				
“Female”-5 Joinpoints	2002–2006	2.57	2.16 ~ 2.97	13.95	< 0.001				
“Female”-5 Joinpoints	2006–2010	3.42	3.02 ~ 3.83	18.52	< 0.001				
“Female”-5 Joinpoints	2010–2015	1.10	0.85 ~ 1.35	9.54	< 0.001				
“Female”-5 Joinpoints	2015–2019	2.91	2.65 ~ 3.16	24.94	< 0.001				
“Both”-5 Joinpoints	1990–1992	0.22	-0.59 ~ 1.03	0.58	0.569	2.35	2.21 ~ 2.48	34.68	< 0.001
“Both”-5 Joinpoints	1992–1995	0.95	0.13 ~ 1.77	2.52	0.026				
“Both”-5 Joinpoints	1995–2007	2.97	2.91 ~ 3.02	115.72	< 0.001				
“Both”-5 Joinpoints	2007–2010	3.60	2.76 ~ 4.44	9.42	< 0.001				
“Both”-5 Joinpoints	2010–2015	1.25	0.99 ~ 1.51	10.47	< 0.001				
“Both”-5 Joinpoints	2015–2019	3.06	2.80 ~ 3.33	25.46	< 0.001				

**Table 2:** Changes in mortality rates of AD and other dementias with high BMI among Chinese residents, 1990–2019 (per 100,000)

	Period	40–44	45–49	50–54	55–59	60–64	65–69	70–74	75–79	80–84	85–89	90–94
Male												
	1990–1994	0.004886	0.033923	0.094701	0.232172	0.490942	0.93317	1.722078	3.615826	10.57888	24.77466	49.62514
	1995–1999	0.005446	0.037793	0.101591	0.247023	0.522527	0.980248	1.793075	3.730649	10.72951	25.46269	50.5593
	2000–2004	0.006578	0.046612	0.124457	0.29876	0.632804	1.20672	2.18308	4.513649	12.40804	29.19942	57.66445
	2005–2009	0.007433	0.053318	0.146583	0.369381	0.780005	1.483537	2.66911	5.557578	14.77144	34.1984	67.57149
	2010–2014	0.008773	0.062355	0.171021	0.428368	0.94202	1.775769	3.173768	6.729473	17.59697	41.05052	83.58457
	2015–2019	0.009786	0.068314	0.185393	0.472756	1.038401	1.968598	3.435294	7.103216	18.5997	44.26456	92.32494
Female												
	1990–1994	0.005581	0.042466	0.130366	0.33204	0.744201	1.461091	2.772386	5.946855	16.73823	38.10718	72.2032
	1995–1999	0.006164	0.048328	0.140157	0.349875	0.797186	1.55474	2.928043	6.151644	17.11862	40.28719	75.68227
	2000–2004	0.007167	0.057591	0.168513	0.413971	0.938059	1.863421	3.570556	7.415695	19.62471	45.55444	86.2005
	2005–2009	0.007705	0.061762	0.194646	0.521027	1.164458	2.278761	4.235381	8.724374	22.46794	50.14496	96.25941
	2010–2014	0.008431	0.069168	0.214501	0.590384	1.403754	2.707011	5.048788	10.43627	26.17921	58.72252	113.0039
	2015–2019	0.009023	0.071467	0.224446	0.620652	1.469762	2.886517	5.376566	10.98439	27.19181	61.57539	120.5462

### Age-specific-period mortality

**Fig. 2:**
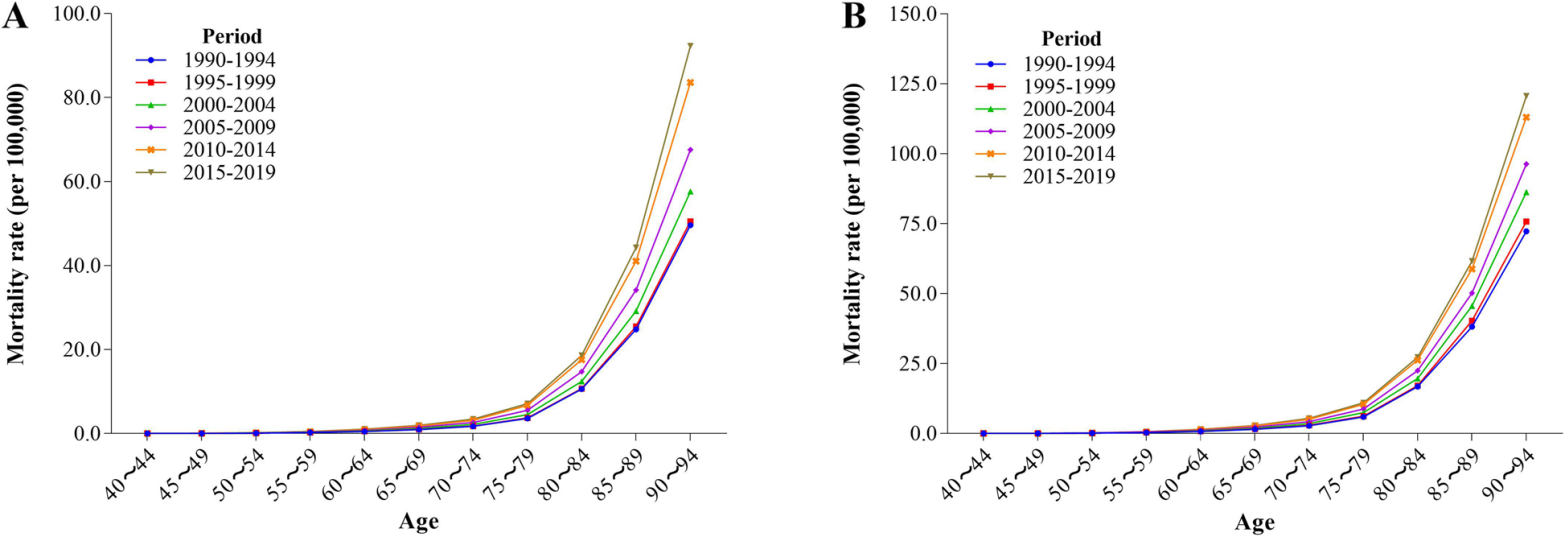
Trends in age-specific mortality of AD and other dementias deaths with high BMI in different periods in China (**A**: Male **B**: Female)

For the six periods 1990 − 1994, 1995 − 1999, 2000 − 2004, 2005 − 2009, 2010 − 2014, and 2015 − 2019, the mortality rates of AD and other dementias with high BMI among Chinese residents exhibited an initial gradual ascent followed by a steeper increase with age. During the 1995–1999 period, the mortality rate for males aged 40–44 increased by approximately 11.46% compared to the 1990–1994 period. Subsequent periods (2000–2004, 2005–2009, 2010–2014, 2015–2019) saw further increases of 20.79%, 13.00%, 18.03%, and 11.55%, respectively. For females in the same age group, there was a rise of about 10.45% in 1995–1999 compared to 1990–1994. Following this, increases of 16.20%, 7.51%, 9.42%, and 7.02% were observed in the subsequent periods (Fig. 2).

### Age-specific-birth cohort mortality

**Fig. 3:**
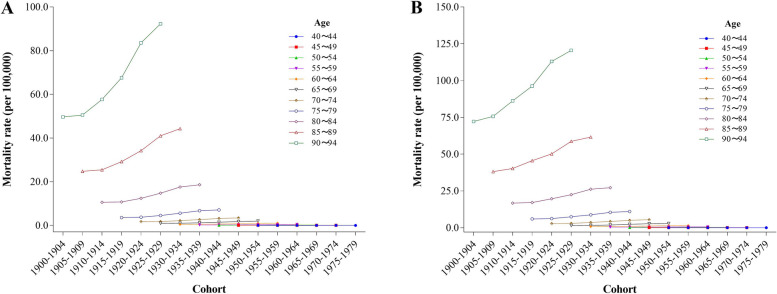
Trends of age-specific mortality of AD and other dementias deaths with high BMI with birth cohorts in China (**A**: Male **B**: Female)

Within distinct birth cohorts, age-specific variations in mortality rates due to AD and other dementias with high BMI are apparent among Chinese residents. For both males (Fig. 3A) and females (Fig. 3B), most age groups present modest variations in mortality rates across cohorts. However, a notable exception is seen in the 80 − 94 years age group, which displays significant fluctuations. This trend is especially prominent for females in the 80 − 94 age range, indicating a pronounced disparity compared to their male counterparts.

### Age-period-cohort model analysis

#### Age effect

**Fig. 4:**
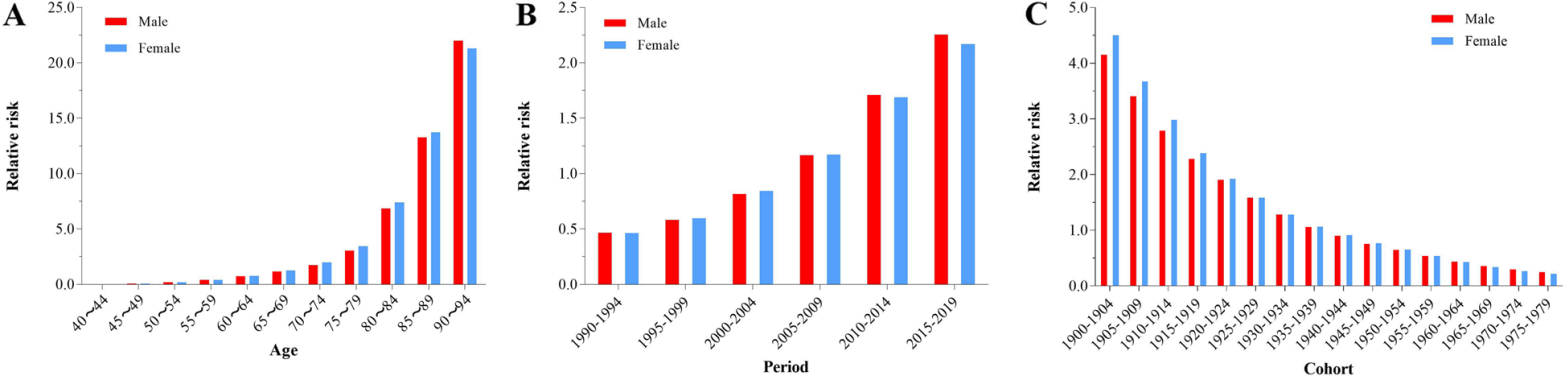
The age, period, and cohort effects on AD and other dementias deaths with high BMI by sex in China (**A**: Age effects **B**: Period effects **C**: Cohort effects)

The RR of mortality from AD and other dementias with high BMI among Chinese residents increased with age (RR increased from 0.01 to 21.98 in males and from 0.01 to 21.33 in females). After adjusting for period and cohort effects, age was found to have a significant impact on mortality from AD and other dementias in those with high BMI. In the age group of 90–94 years, the RRs of mortality for males and females were 18.79 and 16.80 times higher, respectively, compared to those in the 65–69 years age group (Fig. 4A, Supplementary Table 2).

#### Period effect

The mortality RR of AD and other dementias with high BMI among Chinese residents increased over time. The mortality RRs with high BMI for AD and other dementias between 2015 − 2019 for male and female were 4.81 and 4.66 times those in 1990 − 1994, respectively (Fig. 4B, supplementary Table 2).

#### Cohort effect

The mortality RR of AD and other dementias with high BMI in Chinese residents showed a continuous decreasing trend with increasing birth cohort. Compared with 1900 − 1904, the mortality RRs for AD and other dementias with high BMI decreased by 93.98% and 95.17% in male and female in 1975 − 1979, respectively (Fig. 4C, Supplementary Table 2).

### Projections on ASMR of AD and other dementias deaths with high BMI

**Fig. 5:**
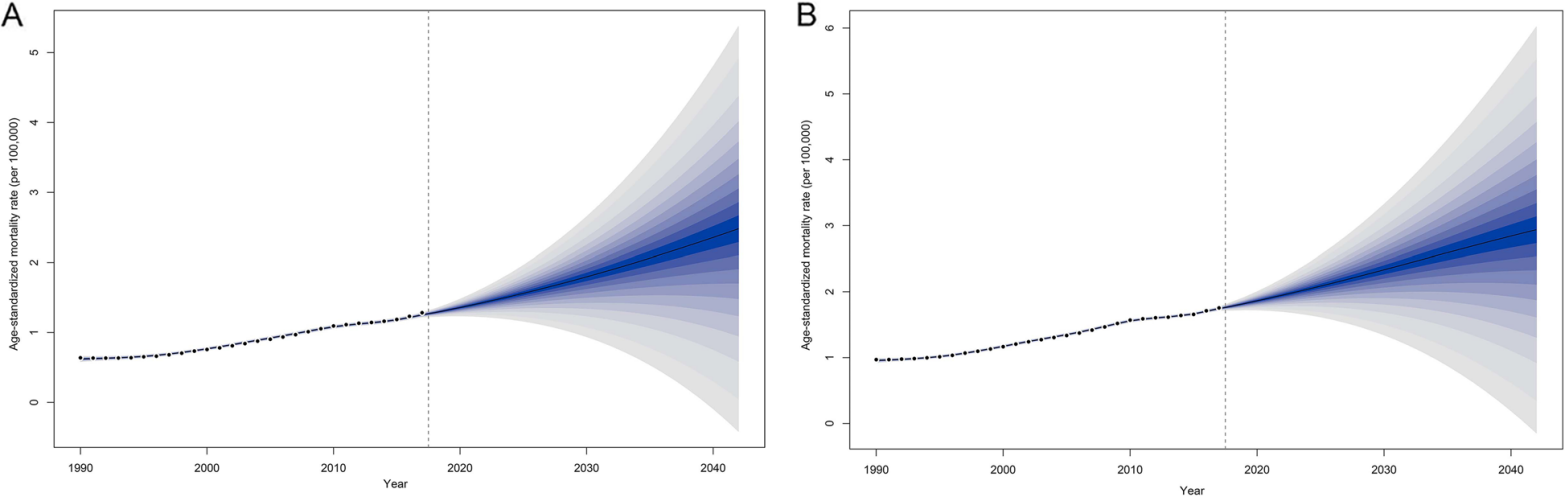
Projections on age-standardized mortality rates of AD and other dementias deaths with high BMI in China (**A**: Male **B**: Female. Note: The shading indicates the range of uncertainty in the model’s predictions, with darker blues showing areas of lower variance and lighter blues indicating higher variance)

From 2020 to 2042, the ASMR of AD and other dementias with high BMI among Chinese residents exhibited an increasing trend, with an increase of 82.35% [from 1.36 (1.32, 1.40) to 2.49 (1.49, 3.49) per 100,000] in males and an increase of 58.06% [from 1.86 (1.82, 1.91) to 2.94 (1.88, 4.03) per 100,000] in females (Fig. 5, Supplementary Table 3).

## Discussion

Our research underscores a steady increase in mortality rates associated with AD and other dementias in high BMI individuals in China from 1990 to 2019. Notably, females exhibited higher rates than males. Joinpoint regression models showed the ASMR for males rose by an average of 2.70% annually from 1990 − 2019 (*P* = 0.008), and for females, it increased by 2.29% per year (*P* < 0.001). This uptrend correlates with both the aging Chinese population and the surge in high BMI. However, the impact of an aging population is more pronounced. For reference, a study by Ma et al. [25] highlighted the growing rates of high BMI in China, with the prevalence of being overweight jumping from 26.6% in 1993 to 41.3% in 2015. Furthermore, our data is consistent with a survey indicating higher mortality rates in females with AD and other dementias than in males [26]. Multiple factors, such as demographic aging, increased life expectancy, expanded healthcare coverage, and updated diagnostic standards, contribute to these observed trends.

Age was a pivotal factor in our study, emphasizing that the risk ratio of mortality from AD and other types of dementia increased among Chinese residents with higher BMI as they aged [27]. According to the 7th National Census of China (2020), the proportion of Chinese older individuals aged 65 years old and above was 13.5%, in 2020, which indicates that China is an aging society [28]. This suggests that as the elderly population continues to increase, the social burden of AD and other dementias will also increase.

The period effect is the risk that a particular social environment or natural condition will lead to a change in mortality after controlling for age and cohort effects. The period effect shows that the RR of mortality from AD and other dementias with high BMI among Chinese residents tended to increase over time. This trend may be related to the development of the Chinese economy, which has brought about a change in people’s perception of food consumption. From 1949 to 1992, the Chinese diet underwent a shift from a coarse-grain and carbohydrate-based diet to one with fewer carbohydrates and a significant increase in high-fat, animal-derived foods [29]. In addition, the decline in physical activity brought about by motorized transportation and changes in occupation, such as shifts from physically demanding jobs to more sedentary ones, also played a role [30]. The rise in high BMI, diabetes, and other diseases can be attributed to increased fat intake and the reduced energy expenditure.

Cohort effects are defined as exposures to different factors for different birth cohorts after controlling for age and period effects. The cohort effect revealed that the RR of mortality from AD and other dementias with high BMI in Chinese residents showed a downward trend with birth cohort. Analysis of the declining birth cohort effect in Chinese residents may be explained by knowledge of AD and other dementia risk factors, and prevention strategies among younger generations, significantly higher awareness of disease prevention, and increased educational attainment. Kuo et al. [31] found that early education stimulates the brain to build cognitive reserves, and therefore, higher education may reduce the risk of dementia in later life. Advances in medical and health care, and public health interventions may also make interventions for AD and other dementias more effective in delaying cognitive decline, and thus, reducing the burden on the patient’s family and society.

Our projections showed that the ASMR for AD and other dementias with high BMI among Chinese residents will rise from 2020 − 2042. It is important to note that the rise is expected to be higher in males than in females, while the overall level remains lower in males compared to females. This forecast may likely be due to a combination of rapidly changing lifestyle factors, increased healthcare utilization and diagnosis in men, and a lower initial prevalence making any rise more pronounced [32]. As China continues to modernize, the health burden caused by high BMI may continue to increase in the future. China requires vigorous interventions for the prevention of high BMI in adults.

The shortcomings of this study include the following: Firstly, data sourced from GBD2019 and analyzed with mathematical models may introduce biases, including potential overfitting and data source variability. Furthermore, the use of Joinpoint regression models, which assume uniform data trends, may not adequately capture non-linear interactions, potentially oversimplifying complex disease dynamics. Thirdly, and crucially, there exists a potential discrepancy between our model’s predictions and actual future outcomes. This concern arises from the inherent uncertainties involved in projecting future trends based on historical data. Finally, the absence of direct comparison between individuals with high BMI and those of normal weight restricts our insight into the nuanced relationship between BMI categories and disease outcomes.

For future research, including data for both high BMI and normal-weight individuals is recommended to allow for a more comprehensive analysis. Exploring alternative statistical models that account for non-linear relationships and interactions between risk factors would also enhance our analysis.

## Conclusion

There was an overall increasing trend in mortality from AD and other dementias with high BMI in China from 1990 − 2019. The age effect showed that the mortality RR of AD and other dementias with high BMI in Chinese residents increased with age. The period effect showed an increasing trend in mortality RR over time, and the cohort effect showed a decreasing trend in mortality RR over the cohort. Our projections suggest that the ASMR will rise more for male residents than females in China from 2020 − 2042. With the aging of the Chinese population and the increasing prevalence of high BMI, it is crucial to implement intervention policies as early as possible and prioritize preventive measures for AD. This will help reduce the burden of dementia in Chinese society, particularly among the elderly.

### Supplementary Information

**Supplementary Material 1.**

## Data Availability

The original contributions presented in the study are included in the article/Supplementary Material, further inquiries can be directed to the corresponding author.
