# Supplementary material for: Long-term trends in Alzheimer’s disease and other dementias deaths with high body mass index in China from 1990 to 2019, and projections up to 2042
Source: Arch Public Health. 2024 Mar 26;82:42. doi: 10.1186/s13690-024-01273-w (PMC10964531; doi:10.1186/s13690-024-01273-w)
Supplement: Supplementary file 1 — Supplementary Material 1. [file 13690_2024_1273_MOESM1_ESM.docx]

Supplementary Table 1. Confidence Intervals for APC and AAPC in AD and Other Dementias

| Class | Lower 95% CI | Upper 95% CI |
| --- | --- | --- |
| APC | $100\{\exp\left( b-s*t_{d}^{-1}\left( 1-\frac{\alpha}{2} \right) \right)-1\}$ | $100\{\exp\left( b+s*t_{d}^{-1}\left( 1-\frac{\alpha}{2} \right) \right)-1\}$ |
| AAPC | $\{\exp\left[ \log\left( \left( \frac{AAPC}{100} \right)+1 \right)-z_{1-\frac{\alpha}{2\sqrt{\sum\breve{\omega}_{i}^{2}\breve{\sigma}_{i}^{2}}}} \right]-1\}\times100$ | $\{\exp\left[ \log\left( \left( \frac{AAPC}{100} \right)+1 \right)+z_{1-\frac{\alpha}{2\sqrt{\sum\breve{\omega}_{i}^{2}\breve{\sigma}_{i}^{2}}}} \right]-1\}\times100$ |
| Note: APC: ***b*** is the estimated slope, ***s*** is the standard error for the slope listed in the output, ***d*** is the degrees of freedom, and $t_{d}^{-1}$-1(q) is the ***q^th^*** quantile of a t distribution with d degrees of freedom. AAPC: ***Ζ***_α_ is the ***α***^th^ quantile of the standard normal distribution, and $\breve{\omega}_{i}^{2}$ denotes the estimate variance of ***b_i_*** obtained from the fit of the joinpoint model. | | |

Supplementary Table 2. APC modeling results of AD and other dementias deaths with high BMI by sex in China.

| Factors | Males | China deaths (Coef) | | *Z* | *P* | RR | Females | China deaths (Coef) | | *Z* | *P* | RR |
| --- | --- | --- | --- | --- | --- | --- | --- | --- | --- | --- | --- | --- |
|  |  | 95% CI | |  |  |  |  | 95% CI | |  |  |  |
|  |  | Lower | Upper |  |  |  |  | Lower | Upper |  |  |  |
| Age |  |  |  |  |  |  |  |  |  |  |  |  |
| 40~44 | -4.20 | -13.34 | 4.95 | -0.90 | 0.369 | 0.01 | -4.44 | -13.32 | 4.44 | -0.98 | 0.327 | 0.01 |
| 45~49 | -2.43 | -6.01 | 1.15 | -1.33 | 0.183 | 0.09 | -2.58 | -5.91 | 0.75 | -1.52 | 0.129 | 0.08 |
| 50~54 | -1.62 | -4.24 | 1.01 | -1.20 | 0.228 | 0.20 | -1.67 | -4.06 | 0.72 | -1.37 | 0.171 | 0.19 |
| 55~59 | -0.89 | -2.96 | 1.18 | -0.84 | 0.398 | 0.41 | -0.90 | -2.78 | 0.98 | -0.94 | 0.348 | 0.41 |
| 60~64 | -0.31 | -1.99 | 1.38 | -0.36 | 0.722 | 0.73 | -0.25 | -1.78 | 1.28 | -0.32 | 0.746 | 0.78 |
| 65~69 | 0.16 | -1.22 | 1.53 | 0.22 | 0.825 | 1.17 | 0.24 | -1.02 | 1.49 | 0.37 | 0.709 | 1.27 |
| 70~74 | 0.56 | -0.54 | 1.67 | 1.00 | 0.319 | 1.75 | 0.69 | -0.32 | 1.71 | 1.34 | 0.181 | 1.99 |
| 75~79 | 1.12 | 0.24 | 1.99 | 2.50 | 0.013 | 3.06 | 1.24 | 0.42 | 2.05 | 2.98 | 0.003 | 3.46 |
| 80~84 | 1.93 | 1.20 | 2.65 | 5.22 | <0.001 | 6.89 | 2.00 | 1.31 | 2.69 | 5.72 | <0.001 | 7.39 |
| 85~89 | 2.59 | 1.88 | 3.29 | 7.19 | <0.001 | 13.33 | 2.62 | 1.94 | 3.30 | 7.58 | <0.001 | 13.74 |
| 90~94 | 3.09 | 2.28 | 3.91 | 7.42 | <0.001 | 21.98 | 3.06 | 2.28 | 3.84 | 7.67 | <0.001 | 21.33 |
| Period |  |  |  |  |  |  |  |  |  |  |  |  |
| 1990-1994 | -0.76 | -1.56 | 0.04 | -1.86 | 0.063 | 0.47 | -0.77 | -1.50 | -0.04 | -2.07 | 0.039 | 0.46 |
| 1995-1999 | -0.54 | -1.04 | -0.04 | -2.11 | 0.034 | 0.58 | -0.52 | -0.97 | -0.06 | -2.23 | 0.026 | 0.59 |
| 2000-2004 | -0.20 | -0.46 | 0.05 | -1.57 | 0.116 | 0.82 | -0.17 | -0.39 | 0.04 | -1.55 | 0.120 | 0.84 |
| 2005-2009 | 0.15 | -0.10 | 0.41 | 1.20 | 0.230 | 1.16 | 0.16 | -0.06 | 0.37 | 1.44 | 0.149 | 1.17 |
| 2010-2014 | 0.54 | 0.05 | 1.02 | 2.16 | 0.031 | 1.72 | 0.52 | 0.08 | 0.97 | 2.3 | 0.021 | 1.68 |
| 2015-2019 | 0.81 | 0.05 | 1.58 | 2.08 | 0.037 | 2.25 | 0.77 | 0.07 | 1.48 | 2.15 | 0.032 | 2.16 |
| Cohort |  |  |  |  |  |  |  |  |  |  |  |  |
| 1900-1904 | 1.42 | -0.19 | 3.03 | 1.73 | 0.083 | 4.14 | 1.50 | -0.06 | 3.07 | 1.89 | 0.059 | 4.48 |
| 1905-1909 | 1.23 | -0.18 | 2.64 | 1.7 | 0.088 | 3.42 | 1.30 | -0.09 | 2.70 | 1.83 | 0.068 | 3.67 |
| 1910-1914 | 1.03 | -0.24 | 2.30 | 1.58 | 0.113 | 2.80 | 1.09 | -0.19 | 2.37 | 1.67 | 0.095 | 2.97 |
| 1915-1919 | 0.82 | -0.37 | 2.02 | 1.35 | 0.177 | 2.27 | 0.87 | -0.36 | 2.10 | 1.39 | 0.165 | 2.39 |
| 1920-1924 | 0.64 | -0.55 | 1.84 | 1.05 | 0.292 | 1.90 | 0.65 | -0.58 | 1.89 | 1.04 | 0.298 | 1.92 |
| 1925-1929 | 0.46 | -0.81 | 1.73 | 0.71 | 0.476 | 1.58 | 0.46 | -0.84 | 1.76 | 0.7 | 0.486 | 1.58 |
| 1930-1934 | 0.25 | -1.16 | 1.66 | 0.35 | 0.73 | 1.28 | 0.25 | -1.18 | 1.68 | 0.34 | 0.733 | 1.28 |
| 1935-1939 | 0.06 | -1.56 | 1.67 | 0.07 | 0.943 | 1.06 | 0.06 | -1.54 | 1.66 | 0.08 | 0.939 | 1.06 |
| 1940-1944 | -0.11 | -1.97 | 1.75 | -0.11 | 0.911 | 0.90 | -0.09 | -1.90 | 1.72 | -0.1 | 0.920 | 0.91 |
| 1945-1949 | -0.28 | -2.41 | 1.85 | -0.26 | 0.797 | 0.76 | -0.26 | -2.31 | 1.78 | -0.25 | 0.801 | 0.77 |
| 1950-1954 | -0.44 | -2.85 | 1.98 | -0.35 | 0.723 | 0.64 | -0.43 | -2.73 | 1.87 | -0.37 | 0.715 | 0.65 |
| 1955-1959 | -0.62 | -3.39 | 2.14 | -0.44 | 0.658 | 0.54 | -0.62 | -3.24 | 1.99 | -0.47 | 0.640 | 0.54 |
| 1960-1964 | -0.82 | -4.10 | 2.45 | -0.49 | 0.622 | 0.44 | -0.85 | -3.92 | 2.23 | -0.54 | 0.590 | 0.43 |
| 1965-1969 | -1.03 | -5.26 | 3.20 | -0.48 | 0.633 | 0.36 | -1.09 | -5.08 | 2.90 | -0.53 | 0.594 | 0.34 |
| 1970-1974 | -1.22 | -7.74 | 5.30 | -0.37 | 0.714 | 0.30 | -1.32 | -7.69 | 5.04 | -0.41 | 0.684 | 0.27 |
| 1975-1979 | -1.39 | -21.89 | 19.10 | -0.13 | 0.894 | 0.25 | -1.53 | -22.60 | 19.54 | -0.14 | 0.887 | 0.22 |
| Deviance | 0.004 |  |  |  |  |  | 0.005 |  |  |  |  |  |
| AIC | 3.49 |  |  |  |  |  | 3.78 |  |  |  |  |  |
| BIC | -150.68 |  |  |  |  |  | -150.66 |  |  |  |  |  |

Note: Coef: Coefﬁcient; AIC: Akaike Information Criterions; BIC: Bayesian Information Criterions.

Supplementary Table 3. Projected ASMR of AD and other dementias deaths with high BMI by sex in China, 2020 to 2042 (1/100,000)

| Year | Males | | | Females | | |
| --- | --- | --- | --- | --- | --- | --- |
|  | Q25 | Q50 | Q75 | Q25 | Q50 | Q75 |
| 2020 | 1.32 | 1.36 | 1.40 | 1.82 | 1.86 | 1.91 |
| 2021 | 1.34 | 1.40 | 1.45 | 1.84 | 1.91 | 1.97 |
| 2022 | 1.36 | 1.44 | 1.51 | 1.86 | 1.95 | 2.04 |
| 2023 | 1.38 | 1.48 | 1.57 | 1.89 | 2.00 | 2.10 |
| 2024 | 1.41 | 1.52 | 1.63 | 1.91 | 2.04 | 2.18 |
| 2025 | 1.42 | 1.56 | 1.70 | 1.93 | 2.09 | 2.25 |
| 2026 | 1.44 | 1.61 | 1.77 | 1.94 | 2.14 | 2.33 |
| 2027 | 1.46 | 1.65 | 1.85 | 1.96 | 2.19 | 2.41 |
| 2028 | 1.48 | 1.70 | 1.92 | 1.97 | 2.24 | 2.50 |
| 2029 | 1.49 | 1.75 | 2.01 | 1.98 | 2.29 | 2.59 |
| 2030 | 1.50 | 1.80 | 2.09 | 1.99 | 2.34 | 2.68 |
| 2031 | 1.51 | 1.85 | 2.19 | 2.00 | 2.39 | 2.78 |
| 2032 | 1.52 | 1.90 | 2.28 | 2.01 | 2.44 | 2.88 |
| 2033 | 1.53 | 1.96 | 2.38 | 2.01 | 2.49 | 2.98 |
| 2034 | 1.54 | 2.01 | 2.49 | 2.01 | 2.55 | 3.09 |
| 2035 | 1.54 | 2.07 | 2.59 | 2.00 | 2.60 | 3.20 |
| 2036 | 1.54 | 2.12 | 2.71 | 1.99 | 2.65 | 3.31 |
| 2037 | 1.54 | 2.18 | 2.83 | 1.98 | 2.70 | 3.42 |
| 2038 | 1.54 | 2.24 | 2.95 | 1.97 | 2.75 | 3.54 |
| 2039 | 1.53 | 2.30 | 3.08 | 1.95 | 2.80 | 3.66 |
| 2040 | 1.52 | 2.36 | 3.21 | 1.93 | 2.85 | 3.78 |
| 2041 | 1.50 | 2.43 | 3.35 | 1.90 | 2.90 | 3.90 |
| 2042 | 1.49 | 2.49 | 3.49 | 1.88 | 2.95 | 4.03 |
